# Supplementary Oral Anamorelin Mitigates Anorexia and Skeletal Muscle Atrophy Induced by Gemcitabine Plus Cisplatin Systemic Chemotherapy in a Mouse Model

**DOI:** 10.3390/cancers12071942

**Published:** 2020-07-17

**Authors:** Makito Miyake, Shunta Hori, Yoshitaka Itami, Yuki Oda, Takuya Owari, Tomomi Fujii, Sayuri Ohnishi, Yosuke Morizawa, Daisuke Gotoh, Yasushi Nakai, Satoshi Anai, Kazumasa Torimoto, Nobumichi Tanaka, Kiyohide Fujimoto

**Affiliations:** 1 Department of Urology, Nara Medical University School of Medicine, Nara 634-8522, Japan; horimaus@gmail.com (S.H.); y.itami.324@gmail.com (Y.I.); ykoda2016@gmail.com (Y.O.); tintherye@gmail.com (T.O.); sayuri3@naramed-u.ac.jp (S.O.); tigers.yosuke@gmail.com (Y.M.); dgotou@gmail.com (D.G.); nakaiyasusiuro@live.jp (Y.N.); sanai@naramed-u.ac.jp (S.A.); torimoto@naramed-u.ac.jp (K.T.); sendo@naramed-u.ac.jp (N.T.); kiyokun@naramed-u.ac.jp (K.F.); 2 Department of Diagnostic Pathology, Nara Medical University School of Medicine, Nara 634-8521, Japan; fujiit@naramed-u.ac.jp; 3 Department of Prostate Brachytherapy, Nara Medical University School of Medicine, 840 Shijo-cho, Kashihara, Nara 634-8522, Japan

**Keywords:** chemotherapy, anamorelin, urothelial cancer, bladder neoplasm, appetite loss, muscle atrophy, sarcopenia, mucositis, gemcitabine, cisplatin

## Abstract

Chemotherapy-induced adverse effects can reduce the relative dose intensity and quality of life. In this study, we investigated the potential benefit of supplementary anamorelin and 5-aminolevulinic acid (5-ALA) as preventive interventions against a gemcitabine and cisplatin (GC) combination chemotherapy-induced adverse effects in a mouse model. Non-cancer-bearing C3H mice were randomly allocated as follows and treated for 2 weeks—(1) non-treated control, (2) oral anamorelin alone, (3) oral 5-ALA alone, (4) gemcitabine and cisplatin (GC) chemotherapy, (5) GC plus anamorelin, and (6) GC plus 5-ALA. GC chemotherapy significantly decreased body weight, food intake, skeletal muscle mass and induced severe gastric mucositis, which resulted in decreased ghrelin production and blood ghrelin level. The supplementation of oral anamorelin to GC chemotherapy successfully mitigated decrease of food intake during the treatment period and body weight loss at day 8. In addition, analysis of the resected muscles and stomach revealed that anamorelin suppressed chemotherapy-induced skeletal muscle atrophy by mediating the downregulation of forkhead box protein O-1 (FOXO1)/atrogin-1 signaling and gastric damage. Our findings suggest the preventive effect of anamorelin against GC combination chemotherapy, which was selected for patients with some types of advanced malignancies in clinical practice.

## 1. Introduction

Gemcitabine and cisplatin chemotherapy (GC) is an active combination in the treatment of advanced/metastatic malignancies, such as urothelial carcinoma (UC) [1], non-small cell lung cancer [2], and biliary tract cancer [3]. One of the biggest clinical issues is that most patients receiving chemotherapy, particularly cisplatin, suffer from adverse effects, including anorexia, vomiting, gastrointestinal damage, muscle loss, myelosuppression, nephrotoxicity, and ototoxicity, which can limit the effective dosage of chemotherapy drugs [4,5,6,7]. Therefore, it is desirable to develop effective supplementary drugs or interventions to mitigate chemotherapy-induced adverse effects.

Chemotherapy-induced acute nausea and vomiting involve serotonin secretion from enterochromaffin cells [5,8]. Administration of a 5-HT3-receptor antagonist ameliorates these gastrointestinal disorders in the acute stage. In addition, anorexia and mucositis are known to be other adverse events induced by chemotherapy. Although the mechanisms underlying the chemotherapy-induced anorexia remains to be fully elucidated, ghrelin is one of the key gastrointestinal hormones, which regulate feeding and might serve as therapeutic targets for anorexia. Ghrelin is a 28-amino acid peptide secreted from the stomach and behaves as an endogenous ligand for the ghrelin receptor [9]. Administration of ghrelin in cancer-associated cachexia can promote gastric acid production and enhance appetite, improve quality of life (QOL), and inhibit pro-inflammatory cytokines [10,11]. Although ghrelin is a potentially useful supplement for minimizing chemotherapy-induced adverse effects, its short half-life (approximately 30 min) is a significant drawback. To overcome this drawback, an orally active ghrelin mimetic, anamorelin (ONO-7643; RC-1291), a ghrelin receptor agonist, was developed and tested for various types of malignancies [11]. Similar to cachexia status, cisplatin-based chemotherapy decreased plasma ghrelin and food intake in patients with esophageal cancer [8]. Furthermore, a Japanese prospective, randomized, placebo-controlled study targeting esophageal cancer confirmed that co-administration of intravenous infusions of synthetic human ghrelin (3 μg/kg) twice daily for one week with cisplatin-based neoadjuvant chemotherapy suppressed chemotherapy-induced anorexia, nausea, and vomiting [12].

5-aminolevulinic acid (5-ALA) is distributed ubiquitously in mammalian cells and is a precursor of tetrapyrole compounds such as heme, which is essential in aerobic energy metabolism and the electron transport system [13]. Previous studies showed that 5-ALA exerts a broad range of cytoprotective effects against cisplatin-induced nephrotoxicity, hypoxia-induced cardiomyocyte injury, and radiotherapy-induced damage of the surrounding normal organs, partly through its antioxidant capabilities [13,14,15]. Based on preclinical evidence, several prospective trials are ongoing to evaluate the potential clinical effects of oral 5-ALA phosphate with sodium ferrous citrate in unresectable gastric cancer (trial ID: UMIN000024642), localized prostate cancer (jRCTs051190077), mitochondrial disease (JMA-IIA00358), Alzheimer’s disease (jRCTs041180135), and autism spectrum disorder (jRCTs051190017). In addition to anamorelin, in this study we investigated the potential of 5-ALA for preventing the negative effects induced by systemic chemotherapy.

To date, there are no reports that have evaluated the potential use of supplementary oral anamorelin and 5-ALA for the GC chemotherapy regimen, which is frequently administered to patients with advanced UC. Here, we investigated their effects against GC chemotherapy-induced adverse events, including anorexia, decreased food intake, body weight loss, and skeletal muscle atrophy, and the underlying molecular mechanisms, using a rodent model.

## 2. Results

**Figure 1:**
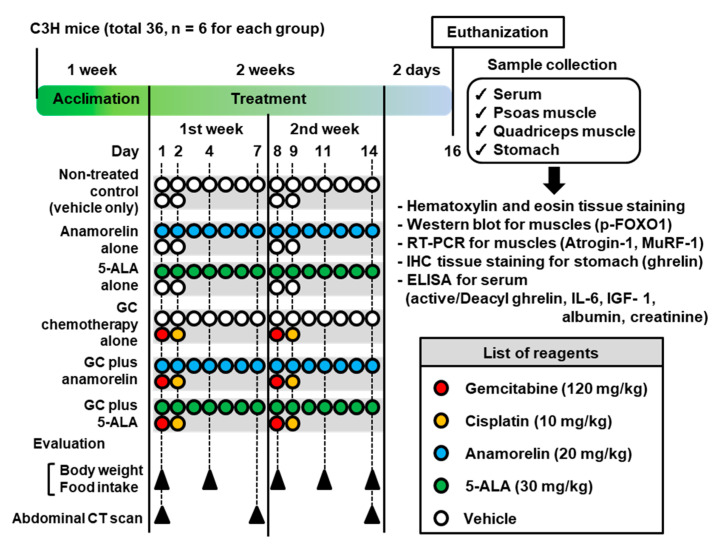
Experimental design of this study. Abbreviations: 5-ALA—5-aminolevulinic acid; GC—gemcitabine and cisplatin combination chemotherapy; CT—computed tomography; p-FOXO1—phospho-forkhead box protein O-1; RT–PCR—reverse transcriptase polymerase chain reaction; IHC—immunohistochemistry; MuRF1—muscle RING finger 1; ELISA—enzyme-linked immunosorbent assay; IL-6—interleukin-6; IGF-1—insulin-like growth factor-1.

The experimental design, treatment, tissue collection, and analysis methods are depicted in Figure 1.

### 2.1. Time-Course Changes in Body Weight and Food Intake During Treatment

Changes in body weight and daily food intake are summarized in Table 1 and Table 2, respectively. The values were compared at each time-point with the non-treated control group or the GC alone group, as indicated. Treatment with anamorelin alone and 5-ALA alone did not affect body weight or food intake throughout the 2-week treatment, whereas GC chemotherapy led to significant body weight loss, mainly owing to decreased food intake. The daily food intake was half to one-third that of the non-treated control, during the treatment. Evaluation of oral 5-ALA and anamorelin supplementation with GC chemotherapy demonstrated that the latter significantly mitigated body weight loss (*p* = 0.032 at day 8; vs GC alone) and decrease of food intake (*p* = 0.002–0.006 during the treatment; vs GC alone). Although anamorelin supplementation mitigated GC-induced decrease of food intake throughout the treatment, the difference between GC chemotherapy alone and GC plus anamolerin at days 1, 4, 11, and 14 did not reach significance.

### 2.2. Preventive Effect of Anamorelin Against Chemotherapy-Induced Skeletal Muscle Atrophy

**Figure 2:**
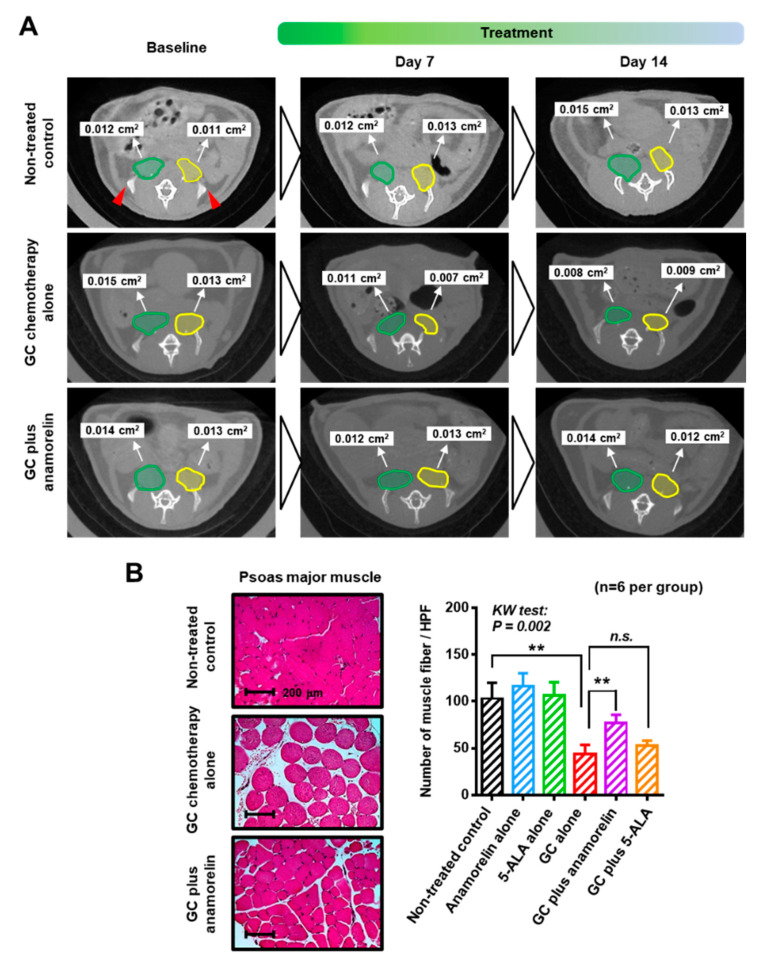
Changes in skeletal muscles owing to chemotherapy. (**A**) Measurement of the cross-sectional area of the bilateral psoas major muscle at the level of the top of iliac bones (red triangles) was performed using a combination of computed tomography and NIH Image J. Right (green ellipse) and left (yellow ellipse) psoas major muscles were measured separately and summed. (**B**) Representative hematoxylin and eosin staining images are shown for the non-treated control, gemcitabine and cisplatin chemotherapy (GC) alone, and GC plus (continued) anamorelin groups. Original magnification: 200×. Quantifying HE-stained muscle mass was performed with Image J. The number of muscle fiber per high power field (HPF) was counted and compared among the treatment groups by the Kruskal-Wallis test and the post-hoc test (Dunn test). Abbreviations: 5-ALA—5-aminolevulinic acid; GC—gemcitabine and cisplatin combination chemotherapy; CT—computed tomography; HE—hematoxylin & eosin.

The cross-sectional area (cm^2^) of psoas major muscle (PMM) of each treatment group was monitored at baseline, day 7, and day 14 (Figure 2A and Table 3). Similar to the evaluation of body weight and food intake, PMM was not affected by treatment with anamorelin alone and 5-ALA alone. Significant atrophic changes in PMM were observed at days 7 and 14, between the non-treated control (0.024 and 0.025 cm^2^, respectively) and the GC chemotherapy (0.018 and 0.018 cm^2^, respectively). This negative effect induced by the chemotherapy was ameliorated by supplementary anamorelin (0.022 and 0.023 cm^2^, respectively), but not by 5-ALA (0.019 and 0.019 cm^2^, respectively). Pathological examination based on hematoxylin & eosin (HE)-stained sections showed muscle fiber atrophy and fat deposition, which was significantly alleviated by supplementary anamorelin (Figure 2B). The numbers of muscle fiber in HE-stained sections was 103 ± 17, 43 ± 11, and 77 ± 9 in the non-treated control, GC chemotherapy, and GC plus anamorelin, respectively.

**Figure 3:**
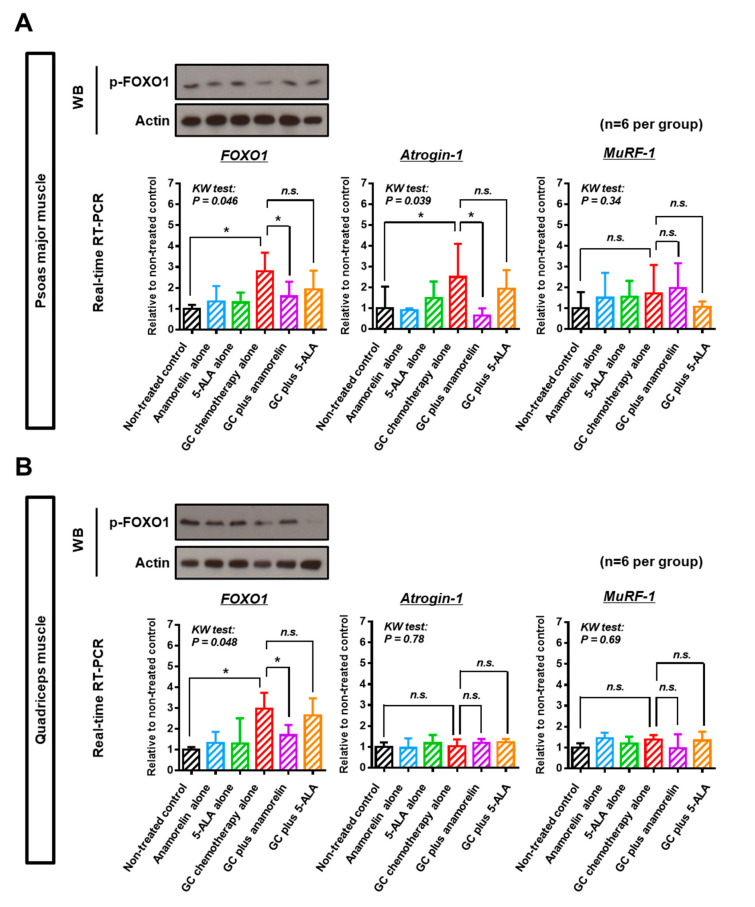
Three muscle atrophy-related genes in the psoas major muscle and quadriceps muscle of treated mice. Phosphorylation level of FOXO1 (p-FOXO1) and gene expressions of total FOXO1, atrogin-1, and MuRF-1 in the psoas major muscle (**A**) and quadriceps muscle (**B**) were determined using Western blotting and quantitative real-time RT-PCR. In the Western blotting analysis, anti-actin antibody was used as an internal loading control. Western blot analysis was performed to evaluate phosphorylation level of FOXO1. Actin served as a loading control. The fold change of the stained muscle mass of each treatment was expressed by relative value to the non-treated control (set to 1). Representative blots are shown in this figure and the results were confirmed in three independent experiments. In the analysis of RT-PCR, the level of each treatment group was expressed as the relative value after normalization with the beta-actin expression level. Marked attention was paid to the relationship among the non-treated control, gemcitabine/cisplatin (GC) chemotherapy alone, and GC plus anamorelin groups. The differences according to the type of treatment were examined using the Kruskal–Wallis test, followed by the Dunn post-hoc test. * *p* < 0.05, ** *p* < 0.01, *n.s.*, not significant.

Next, we explored the molecular mechanism underlying chemotherapy-induced skeletal muscle atrophy and the preventive effect of anamorelin and 5-ALA (Figure 3). As the Forkhead box O (FOXO) family is post-translationally regulated by phosphorylation, phospho-FOXO1 was quantified by Western blot analysis. When phosphorylated, FOXO1 reside in the cytosol and is incapable of entering into the nucleus [16]. Quantitative analysis by densitometry (Figure 3 and Figure S1) revealed that the phosphorylation level of FOXO1 in skeletal muscles was decreased by GC chemotherapy (*p* = 0.03; non-treated control [1.0 ± 0.09] vs. GC chemotherapy [0.55 ± 0.09] in PMM and *p* = 0.03; non-treated control [1.0 ± 0.13] vs. GC chemotherapy [0.65 ± 0.11] in quadriceps muscle). Supplementary anamorelin suppressed dephosphorylation of FOXO1 induced by chemotherapy (*p* = 0.03; GC chemotherapy [0.55 ± 0.09] vs. GC plus anamorelin [1.49 ± 0.36] in PMM and *p* = 0.03; GC chemotherapy [0.65 ± 0.11] vs. GC plus anamorelin [1.36 ± 0.17] in quadriceps muscle). Quantitative reverse transcriptase polymerase chain reaction (RT-PCR) for frozen muscle tissues after treatment suggested that upregulated nuclear FOXO1 and its target *atrogin-1* were involved in atrophy of PMM (Figure 3). In the analysis of quadriceps muscle, upregulated nuclear FOXO1 was observed, but upregulation of *atrogin-1* was not. Supplementary 5-ALA did not affect the expression level of atrogin-1 and MuRF-1 (muscle RING finger 1).

### 2.3. Preventive Effect of Anamorelin Against Chemotherapy-Induced Gastric Mucositis

**Figure 4:**
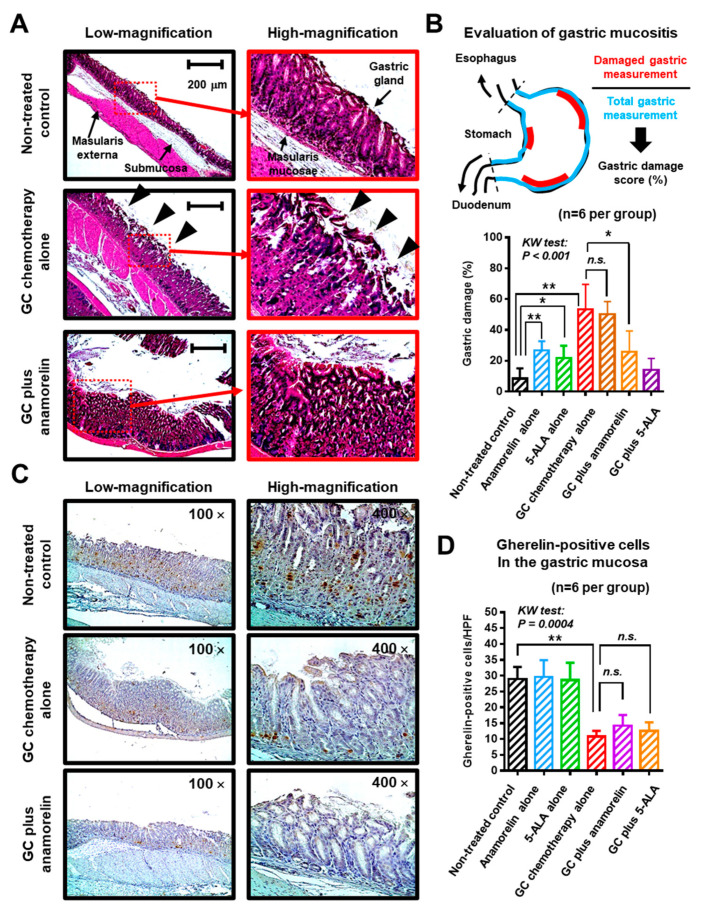
Morphological change and ghrelin expression in gastric mucosa of treated mice. (**A**) Representative morphological images of the gastric wall of non-treated control, gemcitabine/cisplatin (GC) alone, and GC plus anamorelin groups are shown and compared. The panels on the right are magnified images showing the gastric mucosa. GC chemotherapy resulted in substantial changes in the gastric mucosa. The glandular structure was destroyed, resulting in edematous and hemorrhagic mucosa and denuding of the surface layer (black triangles). Thickening of gastric mucosa was found in the GC plus anamorelin group. Original magnification: 200×. (**B**) Mucositis severity was evaluated by the gastric damage score (%), which was determined using the following formula—damaged gastric measurements divided by total measurements in treated mice. (**C**) Immunohistochemical staining for ghrelin expression in the gastric mucosa of mice. Representative images show the distribution of ghrelin-positive cells (brown staining) in the gastric mucosa of mice treated with GC or GC plus anamorelin. (**D**) The number of ghrelin-positive cells/5 high power field (HPF) were counted from specimens of 6 mice and averaged. Values represent the mean ± standard deviation. The differences were examined using the Kruskal–Wallis (KW) test, followed by the Dunn post-hoc test. * *p* < 0.05, ** *p* < 0.01, *n.s.*, not significant.

Chemotherapy damages the epithelium of the oral cavity and the gastrointestinal tract. Chemotherapy-induced mucositis is a serious adverse event associated with chemotherapeutic agents [17,18,19]. Herein, we investigated post-treatment pathological changes in murine gastric mucosa, which is involved in ghrelin production [9]. Organized gastric pits and glands were seen in healthy gastric mucosa of the non-treated controls (Figure 4A). However, GC chemotherapy led to substantial changes in the gastric mucosa. The glandular structure was destroyed, resulting in edematous mucosa and denuding of the surface layer (black triangles). Thickening of gastric mucosa was found in mice in the GC plus anamorelin group. Marked diarrhea was not observed in any treatment group.

Mucositis severity was graded by gastric damage score (%), which was determined using the following formula—damaged gastric measurements divided by total measurements in treated mice (Figure 4B). Treatment with anamorelin alone and 5-ALA alone, induced a certain level of gastric damage because repeated oral administration of highly concentrated drugs using a feeding tube could induce stress to the gastrointestinal tract of mice. GC chemotherapy induced a significantly high gastric damage (53 ± 16%), which was suppressed by oral anamorelin (26 ± 14%), but not by 5-ALA (50 ± 8%). Next, we performed immunohistochemical staining for ghrelin expression in the gastric mucosa. Representative images show diffuse distribution of ghrelin-positive cells in the gastric mucosa (Figure 4C). Notably, GC chemotherapy decreased ghrelin production from the stomach. Similar to the result of gastric mucositis, deterioration of ghrelin production from gastric mucosa was not suppressed by oral anamorelin and 5-ALA (Figure 4D).

### 2.4. Blood Levels of Ghrelins and Pro-Inflammatory Cytokines

Ghrelin is a small peptide secreted from gastric cells into circulation, and two isoforms were identified—acylated (active) ghrelin and deacyl (inactive) ghrelin. In addition, we investigated serum levels of IL-6, IGF-1 (insulin-like growth factor), albumin, and creatinine in the treated mice. Of these proteins, deacyl ghrelin, IL-6, albumin, and creatinine were not affected by the GC chemotherapy (Table 4), whereas significant decrease was observed in active ghrelin (*p* = 0.042, vs. non-treated control). The decrease of the IGF-1 level did not reach a significance (*p* = 0.09, vs. non-treated control). The level of IGF-1 was increased with supplementary oral anamorelin (195 ± 39 pg/mL, *p* = 0.034, vs. GC chemotherapy alone). Of note, the level of active ghrelin (2.0 ± 1.1 pg/mL) decreased by GC chemotherapy was not restored to the level of the non-treated control (4.3 ± 1.1 pg/mL) by supplementary oral anamorelin to GC chemotherapy (2.5 ± 0.4 pg/mL). The data of ELISA was consistent with those of immunohistochemical staining for ghrelin expression in the gastric mucosa.

## 3. Discussion

**Figure 5:**
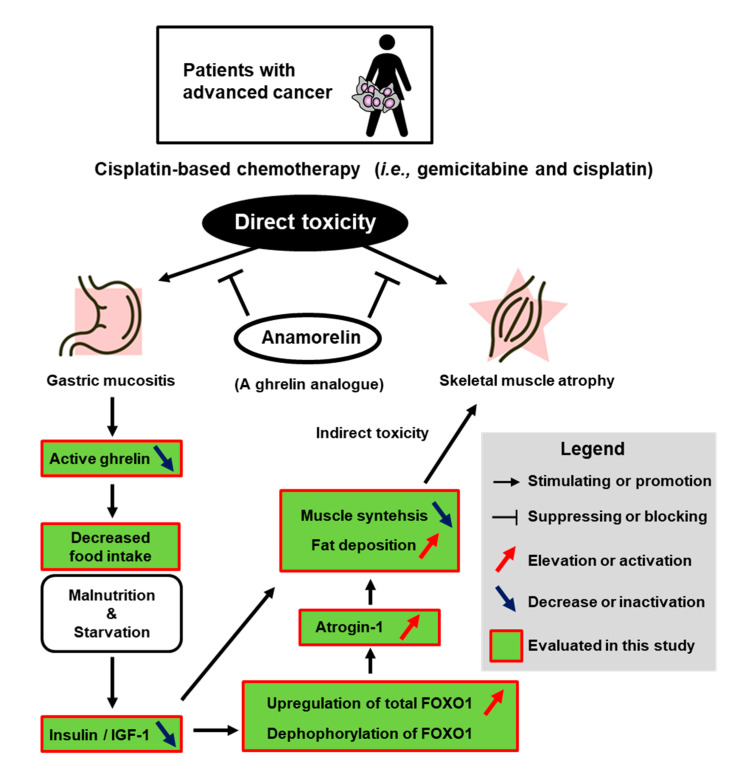
Schema summary of chemotherapy-induced adverse events and the potential of anamorelin intervention. IGF-1—insulin-like growth factor; FOXO1—forkhead box protein O-1.

Our study presented several key findings. Figure 5 depicts a schema summary of chemotherapy-induced adverse events on skeletal muscle atropy and gastric mucositis. First, GC chemotherapy induced anorexia and skeletal muscle atrophy, which might be associated with decreased ghrelin and the phosphorylation of FOXO1 or atrogin-1. Chemotherapeutic drugs could have negative effects on patients and model animals, in terms of functional outcomes like anorexia, muscle atrophy, gastrointestinal tract disorders, decreased QOL, and other types of adverse events. Second, the promising results of mitigating GC chemotherapy-induced negative effects were delivered by a novel non-peptide ghrelin analogue, anamorelin. In our study, no effect of anamorelin was observed on food intake and body weight in healthy non-treated mice (Table 2 and Table 3). Phase I clinical studies demonstrated that administration of anamorelin to healthy adults lead to increases in hunger and caloric intake [20]. We do not have a specific idea for this discrepancy. There is much physiological difference between human and mouse. One possible question would be that non-treated mice were in healthy condition and had sufficient ghrelin in serum. Even if ghrelin analogue was provided to the healthy body, food intake and body weight was not affected by supplementation of anamorelin. Administration of anamorelin could only work in mice whose serum level of ghrelin was low in the mouse model used in our study. Previous clinical and preclinical studies demonstrated that exogenous ghrelin/ghrelin-receptor agonists/ghrelin analogs could exert favorable effects on malignancy-related cachexia and malnutrition [10,11,21]. However, few studies addressed the potential of interventional treatment with exogenous ghrelin/ghrelin-receptor agonists against chemotherapy-induced adverse effects like appetite loss or muscle wasting [12,22].

Chemotherapy-induced adverse effects can cause reduction in relative dose intensity, delay in therapy, and discontinuation of therapy, especially in elderly patients or those with comorbidities. More effort should be made to ameliorate physical stress and psychological pain during and after cancer treatment, and not only for improving oncological outcomes. Chemotherapy-induced nausea, vomiting, and anorexia are the first priority. The American Society of Clinical Oncology Clinical (ASCO) Practice Guideline recommends a four-drug combination of 5-HT3 receptor antagonist, neurokinin 1 receptor antagonist, dexamethasone, and olanzapine (quality of evidence—high; strength of recommendation—strong) for adult patients undergoing treatment with cisplatin and other high-emetic-risk single agents [23]. However, the outcome of this combination was not sufficient because gastrointestinal disorders following chemotherapy can last and persist for several days (delayed-onset) [12,24]. These evidences emphasize on the need for additional interventions providing a long-lasting effect. Treatment with exogenous ghrelin/ghrelin-receptor agonists has been one of the promising approaches for chemotherapy-induced appetite loss/eating disorders/malnutrition. Preclinical and small clinical trials demonstrated that ghrelin administration improved food intake, appetite, nausea, hyperalgesia and cachexia with good tolerability [12,21,25]. A randomized, placebo-controlled phase 2 trial targeting esophageal cancer confirmed that co-administration of intravenous infusions of synthetic human ghrelin with cisplatin-based neoadjuvant chemotherapy suppressed chemotherapy-induced gastrointestinal disorders [12]. However, its short half-life (~30 min) and need for parenteral administration significantly limited its clinical development. Hence, a number of longer-acting, orally bioavailable, synthetic ghrelin analogue were the focus of interest in the field [11,26]. Orally available anamorelin is the most advanced and tested in its development. Two large, international, randomized, double-blind, placebo controlled phase 3 studies in patients with advanced non-small cell lung cancer and cachexia, namely ROMANA 1 and 2, were conducted in the US and Europe [27]. Given anamorelin was well-tolerated and significantly increased lean body mass, anamorelin might be a treatment option for patients with cancer anorexia and cachexia. However, only few studies investigated the clinical potential of supplementary oral anamorelin to mitigate the negative adverse events induced by cancer chemotherapy [25,28].

Although skeletal muscle atrophy is known to be a common side effect of chemotherapy and is associated with poor tolerance to therapy and decreased QOL, the mechanism and appropriate interventions for this complication were not fully characterized [29,30]. A unique aspect of our study was the combined evaluation of skeletal muscle atrophy with appetite loss and gastric mucositis. The main mechanisms and causes underlying chemotherapy-induced muscle atrophy include (1) impaired food intake with reduction in vitamin D, (2) omega 3 fatty acids and protein, (3) reduced physical activity secondary to fatigue, (4) a direct effect of chemotherapy or targeted agents on muscle, and (5) malabsorption secondary to mucositis or treatment-related pancreatic insufficiency [31]. We previously reported a significant loss of skeletal muscle after completion of three-cycle cisplatin-based neoadjuvant chemotherapy in patients with muscle invasive bladder cancer [4]. Chen et al. [22] investigated molecular pathways involving cisplatin-induced muscle wasting using c57/bl6 male mice. Activation of ubiquitin-proteasome-mediated muscle proteolysis through the activation of pro-inflammatory cytokines and downregulation of Akt, myoD, and myogenin resulted in cisplatin-induced muscle atrophy. Additional experiments confirmed that ghrelin prevents muscle wasting and grip strength, eventually leading to prolonged survival.

Both atrogin-1 and MuRF-1 are E3 ubiquitin ligases and predominantly expressed in skeletal muscle, that are involved in the polyubiquitination of target proteins for proteolysis by the 26S proteasome [32]. In our study, chemotherapy-induced muscle atrophy was observed along with the upregulation of atrogin-1 in PMM, which was consistent with the results of previous studies [21,29], while upregulation of MuRF-1 was not seen. The possible explanation is the increase in atrogin-1 and MuRF-1 could be in a transient manner [33,34], making it difficult to accurately measure changes in their expression over time. A similar explanation could be a reason why upregulation of atrogin-1 was not observed, despite upregulated nuclear FOXO1 in the analysis of quadriceps muscle. Moreover, the specific transcription factors that regulate these E3 ubiquitin ligases are not fully elucidated. Although various transcription factors can regulate atrogin-1 and MuRF-1 expression, we focused on FOXO1 in this study. FOXO1 plays a key role in glucose homeostasis and cell cycle progression. This transcription factor was phosphorylated and degraded through the insulin or insulin-like growth factor (IGF) signaling pathway [35]. We found that dephosphorylation of FOXO1 protein, upregulation of total FOXO1 mRNA and decrease in serum IGF-1 concentration were induced by GC chemotherapy. This condition could lead to an inhibition of translocation from nuclear to cytoplasm FOXO1 and upregulation of total FOXO1, resulting in promoting the expression of target genes like atrogin-1 and MuRF-1. Chemotherapy-induced gastrointestinal disorders and anorexia could lead to starvation or low glucose levels, leading to decreased levels of insulin/IGF-1. As a result of phosphorylation of FOXO1, its downstream target gene, atrogin-1, could be upregulated and could stimulate proteolysis and degradation of the skeletal muscle. However, further investigation is needed to prove our speculation on chemotherapy-induced muscle atrophy.

We expected the potential of 5-ALA to act as a cytoprotector from chemotherapeutic drugs. No preventive effect against chemotherapy-induced skeletal muscle atrophy or gastric mucositis was seen in this study. As Terada et al. [14] previously reported that 5-ALA protects the kidney from cisplatin-induced nephrotoxicity in a rat model, we quantified serum creatinine levels after treatment. Our treatment regimen failed to show elevated creatinine levels in mice treated with GC (10 mg/kg of cisplatin once a week for 2 weeks). To exclude all types of nephrotoxicity, in addition to serum creatinine, measurement of blood urea nitrogen and microscopical evaluation of kidney tissues were required. According to a review of rodent models of cisplatin-induced nephrotoxicity [36], multiple doses of 10–40 mg/kg were applied to create mice with kidney injury. Further research is required to assess the preventive effects of anamorelin and 5-ALA against cisplatin-induced nephrotoxicity.

This study had several limitations. First, we did not set up a gemcitabine monotherapy and cisplatin monotherapy regimen. There is much evidence regarding cisplatin-induced gastrointestinal disorders, appetite loss, and skeletal muscle atrophy. We could have included a gemcitabine monotherapy group in this study, however, the combination of gemcitabine and cisplatin is a current standard of care; thus, a more clinically fitted design for patients with advanced malignancies. This study did not include the combination of gemcitabine plus carboplatin, which is an alternatively used regimen for cisplatin-unfit patients. Second, this study performed the animal experiment with young mice and the sample size was small (*n* = 6). This study did not include a measure of muscle function. Moreover, the objective evaluation of appetite loss, nausea or vomiting in rodent model were missing in this study. Third, we did not try a higher dose of 5-ALA (only 30 mg/kg) and did not try the combination of anamorelin and 5-ALA, which could show additive or synergistic preventive effects against chemotherapy-induced adverse events. Fourth, feeding activity and bodily response to chemotherapeutic drugs and supplements differ substantially between humans and mice. Fifth, the abdominal cross-sectional images by CT scan could not be taken at completely the same rostro-caudal levels. This could be a confounding factor because the muscle area might not be the same at each level. Sixth, the blood sampling was conducted only at single point (2 days after completion of the treatment). For example, increased serum level of IGF-1 was not seen in mice treated with anamorelin alone (without GC chemotherapy). We expected that anamorelin would increase the serum IGF-1. This might be explained by possible pharmacokinetic aspects of the anamorelin treatment, in relation to the time-point of blood sampling. The time-point could affect the result of ELISA. Several time-points of blood sampling could provide a different result.

## 4. Materials and Methods

### 4.1. Drugs

Gemcitabine, cisplatin (Tokyo Chemical Industry, Tokyo, Japan), and anamorelin (AdooQ Bioscience, Irvine, CA, USA) were dissolved in sterile water, N,N-dimethylformamide, and dimethyl sulfoxide (Nacalai Tesque, Kyoto, Japan). 5-ALA was supplied by SBI Pharmaceuticals Co., Ltd. (Tokyo, Japan) and dissolved in sterile water. The dosage of gemcitabine, cisplatin, and 5-ALA was determined according to our previous studies [13,36]. Weekly dosage of 120 mg/kg of gemcitabine and 10 mg/kg of cisplatin showed significant antitumor effect in a syngeneic animal model of C3H mice with subcutaneous MBT2 urothelial cancer cells [37]. Daily dosage of 30 mg/kg of 5-ALA showed preventive effect against radiation-induced toxicity on normal organs [13]. The dosage of anamorelin was determined according to the previous study [38].

### 4.2. Animal Experiments

The animal experiment was conducted in compliance with institutional guidelines and regulations after approval from the Committee on Animal Research at the Nara Medical University (reference number: 12659). Specific pathogen-free 5-week-old male C3H mice were purchased from Oriental BioService (Kyoto, Japan). Mice were housed in standard clear plastic cages with free access to food and water and a 12-h light/12-h dark cycle on a controlled environment of temperatures of 65–75 °F and 40–60% humidity. Mice received rodent laboratory chow with a 5.5% fat content. Both treatments and tissue sampling were performed during a light cycle. Non-cancer-bearing mice were used as a treated model because our aim was to investigate the adverse effects purely induced by GC chemotherapy. Tumor progression can lead to cachexia, in which mice suffer from body weight loss, muscle atrophy, and anorexia.

After one-week acclimatization (6-week-old), the mice were allocated randomly into seven groups, as shown in Figure 1: (1) non-treated control (vehicle only), (2) oral anamorelin alone (20 mg/kg/day), (3) oral 5-ALA alone (30 mg/kg/day), (4) intraperitoneal injection with gemcitabine and cisplatin (GC; 120 mg/kg and 10 mg/kg, respectively), (5) GC plus anamorelin, and (6) GC plus 5-ALA. Anamorelin and 5-ALA in 200 μL sterile water was administered to the mice via oral gavage, using a disposable soft catheter tube (FTP-20-30; Primetech, Tokyo, Japan). Six mice were included in each treatment group, and the duration of treatment was 2 weeks. The mice were humanely euthanized 2 days after the completion of treatment. The indicated samples were collected and preserved appropriately until analysis. Blood was collected by cardiac puncture and serum was preserved at −80 °C for the enzyme-linked immunosorbent assay (ELISA). Simultaneously, the muscles and stomach were fixed with 10% neutral-buffered formalin and embedded in paraffin. Sections were cut 3 μm-thick for hematoxylin-eosin (HE) staining Muto Pure Chemicals (Tokyo, Japan) and IHC staining with anti-ghrelin antibody.

### 4.3. Analysis of Psoas Major Muscle (PMM) with Computed Tomography Scanning and Microscopic Examination

Mice were anesthetized with inhalation of isoflurane (FUJIFILM Wako Pure Chemical, Osaka, Japan) and analyzed using an in vivo micro X-ray computed tomography system CosmoScanFX (Rigaku, Tokyo, Japan), without the contrast agents. The tube voltage was set at 90 kV, and the current was constant at 88 μA. Mice were scanned with a field of view of 36 mm and a resolution of 72 μm pixels. The scan duration was 2 min. The exported DICOM image was converted to a single TIFF image format with YAKAMI DICOM Tools version 1.4.5.0. The area of PMM (cm^2^) was analyzed at the level of the top of the bilateral iliac bones (Figure 2A, red triangles), where the outline of PMM was clearly identified (Figure 2A, green and yellow ellipses) and measured by manual tracing, using NIH Image J version 1.52a (National Institutes of Health, Bethesda, MD, USA). The number of muscle fibers per high power field were quantified in HE-stained PMM. Randomly selected five fields was analyzed for each treatment. The investigator (Takuya Owari, Department of Urology, Nara Medical University School of Medicine, Nara, Japan) for quantification of the PMM were blinded to information regarding treatments.

### 4.4. Western Blot Analysis for Phospho-FOXO1

The excised muscles were homogenized and lysed by radioimmunoprecipitation assay (RIPA) buffer containing proteinase inhibitor and phosphatase inhibitor (Sigma-Aldrich, St Louis, MO, USA). Western blotting was performed, as previously described [39]. The phospho-FOXO1 (NB100-81927; Novus Biologicals, Littleton, CO, USA) was used as a primary antibody. Anti-actin antibody (dilution, 1:20,000; clone AC-15, Sigma-Aldrich) was used as an internal loading control. Densitometric assay using the ImageJ software was applied to quantify each band and levels of phospho-FOXO1 was normalized to actin.

### 4.5. Reverse transcriptase polymerase chain reaction (RT-PCR) for muscle atrophy-related genes

The excised muscles were cut into three pieces for HE staining, real-time RT-PCR, and Western blotting. One of three pieces was frozen in DNA/RNA Shield solution (ZYMO Research, Irvine, CA, USA) at −80 °C, prior to analysis. Total RNA was extracted from the frozen tissues of the PMM and the quadriceps muscle, separately, using an RNeasy Mini kit (Qiagen, Valencia, CA, USA), according to the manufacturer’s instructions. Synthesis of cDNA from total RNA was performed using a High Capacity cDNA Reverse Transcription kit (Applied Biosystems, Foster City, CA, USA). Real-time RT-PCR was performed in a reaction tube containing the cDNA, TaqMan primers and probe sets, and TaqMan Fast Universal PCR Master Mix (ThermoFisher Scientific), under the recommended conditions, using a CFX96 Real-time PCR detection system (Bio-Rad, Hercules, CA, USA). CFX Manager™ version 2.1 was used to determine the cycle threshold (Ct). The expression levels of three muscle atrophy-related genes, forkhead box protein O-1 (FOXO1), muscle atrophy F-box (MAFbx; also known as atrogin-1), and muscle RING finger 1 (MuRF1) were quantified using real-time RT-PCR. The TaqMan primers and probe sets are listed in Table S1. The fold change in mRNA expression level of each gene normalized by beta-actin was calculated using the 2−ΔΔCt method. Individual PCRs were performed in triplicates.

### 4.6. Mucosal Damage and Ghreline Expression in the Stomach

The excised stomach was fixed in 10% neutral buffered formalin. Three vertically-cut segments of the fixed stomach were embedded in a paraffin section separately and stained with HE for grading of mucositis severity. HE-stained sections were examined, and three views for each slice were captured with a light microscope (EVOS^®^ FL Auto Cell Imaging System, ThermoFisher Scientific). A total of nine microscopical fields were used to assess a single stomach. Mucositis severity was evaluated by the gastric damage score (%), which was determined using the following formula—damaged gastric measurements divided by total measurements in treated mice. The criteria for damaged gastric mucosa were: (1) architectural disruption of the gastric glands, (2) denuding of the surface layer, and (3) thickening, bleeding, or edema of the mucosa and submucosa. Two experienced investigators (Yuki Oda, Department of Urology, Nara Medical University School of Medicine, Nara, Japan and Tomomi Fujii, Department of Diagnostic Pathology, Nara Medical University School of Medicine, Nara, Japan) carried out the grading without the knowledge of the treatment regimens. The average score was calculated for each treatment group.

Next, IHC staining using paraffin-embedded, formalin-fixed stomach tissues was performed, as previously described [27]. Staining with anti-ghrelin antibody (ab129383, dilution 1:5000; Abcam, Tokyo, Japan) was carried out to evaluate differences among the treated groups. All sections were reviewed by two experienced investigators (Yuki Oda and Tomomi Fujii). The number of ghrelin-positive cells/high power fields (HPF, 400×) was counted and averaged by five HPFs.

### 4.7. ELISA for Blood Proteins

ELISA was performed as previously described [37]. Serum was collected by allowing whole blood to clot at room temperature for 30 min, followed by centrifugation at 1000× *g* for 20 min at 4 °C. Serum samples were preserved in −80 °C until assay. The details of commercially available kits for active ghrelin, deacyl ghrelin, interleukin-6 (IL-6), insulin-like growth factor-1 (IGF-1), albumin, and creatinine are listed in Table S1.

### 4.8. Statistical Analysis

PRISM version 7.00 (GraphPad Software, Inc., San Diego, CA, USA) was used for statistical analysis, plotting the data, and creating graphs. Continuous variables were expressed as the median and interquartile range (IQR) and compared using the Kruskal-Wallis test, followed by the post-hoc test (Dunn test). No data set obtained in this study met the prerequisites for parametric tests. A *p*-value < 0.05 was considered significant.

## 5. Conclusions

GC regimen is frequently selected for patients with advanced solid malignancies in the neoadjuvant chemotherapy setting, adjuvant chemotherapy setting, or palliative setting. This study evaluated the preventive effect of anamorelin against GC chemotherapy-induced adverse events. We found anamorelin was able to mitigate anorexia, gastric mucositis, and skeletal muscle atrophy. This supporting approach might increase health-related quality of life in patients undergoing this combination chemotherapy. In addition, biological approaches including ghrelin staining of stomach and quantification of gene expression of FOXO1 and atrogin-1 in skeletal muscle shed light on the molecular mechanism underlying the uncontrollable adverse events. More efforts to mitigate the toxicity of cancer chemotherapy should be made to optimize longstanding systemic chemotherapy. A prospective clinical trial is mandatory to confirm the full benefit of supplementary oral anamorelin and elucidate the appropriate dose for the management of patients undergoing GC chemotherapy.

## Figures and Tables

**Table 1:** Change in body weight during the treatment.

Treatment Group (n = 6 Per Group)	Body Weight (gram)	Day 1	Day 4	Day 8	Day 11	Day 14
Non-treated control	Mean ± SD	22.0 ± 0.9	22.8 ± 0.8	24.0 ± 0.6	24.2 ± 0.8	24.3 ± 1.0
Anamorelin	Mean ± SD	22.2 ± 0.8	23.4 ± 0.5	24.2 ± 0.8	24.4 ± 0.5	24.2 ± 0.8
	*p* value (vs control)	n.s.	n.s.	n.s.	n.s.	n.s.
5-ALA	Mean ± SD	22.6 ± 0.5	23.4 ± 0.5	23.8 ± 1.6	24.0 ± 0.7	23.6 ± 0.5
	*p* value (vs control)	n.s.	n.s.	n.s.	n.s.	n.s.
GC alone	Mean ± SD	22.2 ± 0.8	21.6 ± 1.3	20.2 ± 1.3	20.0 ± 1.6	22.4 ± 1.1
	*p* value (vs control)	n.s.	n.s.	0.004	0.004	0.028
GC plus anamorelin	Mean ± SD	22.2 ± 0.8	22.8 ± 1.1	22.1 ± 1.2	22.2 ± 2.2	23.9 ± 1.1
	*p* value (vs GC alone)	n.s.	n.s.	0.032	0.071	0.055
GC plus 5-ALA	Mean ± SD	22.2 ± 0.8	22.0 ± 1.0	20.4 ± 1.5	19.2 ± 1.3	21.8 ± 0.8
	*p* value (vs GC alone)	n.s.	n.s.	n.s.	n.s.	n.s.
	*p* value (multiple comparison for six treatment groups)	n.s.	n.s.	0.0014	0.0039	0.011

SD = standard deviation; 5-ALA = 5-aminolevulinic acid; GC = combined chemotherapy of gemicitabine and cisplatin; n.s. = not significant; *p* values are based on the comparison of the values at each treatment time point relative to the non-treated control group or GC alone group with using multiple comparison (the Kruskal-Wallis test) and the post hoc test (Dunn test).

**Table 2:** Daily food intake during the treatment.

Treatment Group (*n* = 6 Per Group)	Daily Food Intake (gram per mouse)	Days 1–4	Days 4–7	Days 7–11	Days 11–14
Non-treated control	Mean ± SD	2.5 ± 0.4	3.6 ± 0.5	3.5 ± 0.5	4.1 ± 0.6
Anamorelin	Mean ± SD	2.7 ± 0.4	3.7 ± 0.6	3.8 ± 0.5	3.8 ± 0.7
	*p* value (vs control)	n.s.	n.s.	n.s.	n.s.
5-ALA	Mean ± SD	2.6 ± 0.6	3.7 ± 0.6	3.4 ± 0.5	3.8 ± 0.9
	*p* value (vs control)	n.s.	n.s.	n.s.	n.s.
GC alone	Mean ± SD	1.2 ± 0.2	1.3 ± 0.2	1.4 ± 0.3	1.7 ± 0.4
	*p* value (vs control)	0.002	0.002	0.002	0.002
GC plus anamorelin	Mean ± SD	1.7 ± 0.2	1.9 ± 0.2	2.2 ± 0.3	2.4 ± 0.3
	*p* value (vs GC alone)	0.002	0.006	0.002	0.006
GC plus 5-ALA	Mean ± SD	1.3 ± 0.3	1.5 ± 0.4	1.7 ± 0.5	2.1 ± 0.3
	*p* value (vs GC alone)	n.s.	n.s.	n.s.	n.s.
	*p* value (multiple comparison for six treatment groups)	n.s.	<0.0001	<0.0001	<0.0001

SD = standard deviation; 5-ALA = 5-aminolevulinic acid; GC = combined chemotherapy of gemicitabine and cisplatin; *p* values are based on the comparison of the values at each treatment time point relative to the non-treated control group or GC alone group with using multiple comparison (the Kruskal-Wallis test) and the post hoc test (Dunn test).

**Table 3:** Time-course change in the psoas major muscle area.

Treatment Group (*n* = 6 Per Group)	Sum of Cross-Sectional Area Bilateral Psoas Major Muscle (cm^2^)	Day 1 (Baseline)	Day 7	Day 14
Non-treated control	Mean ± SD	0.023 ± 0.001	0.024 ± 0.002	0.025 ± 0.002
Anamorelin	Mean ± SD	0.025 ± 0.001	0.025 ± 0.001	0.026 ± 0.001
	*p* value (vs control)	n.s.	n.s.	n.s.
5-ALA	Mean ± SD	0.025 ± 0.002	0.025 ± 0.002	0.026 ± 0.002
	*p* value (vs control)	n.s.	n.s.	n.s.
GC alone	Mean ± SD	0.023 ± 0.002	0.018 ± 0.001	0.018 ± 0.002
	*p* value (vs control)	n.s.	0.022	0.002
GC plus anamorelin	Mean ± SD	0.024 ± 0.002	0.022 ± 0.002	0.023 ± 0.001
	*p* value (vs GC alone)	n.s.	0.004	0.002
GC plus 5-ALA	Mean ± SD	0.025 ± 0.002	0.019 ± 0.002	0.019 ± 0.003
	*p* value (vs GC alone)	n.s.	n.s.	n.s.
	*p* value (multiple comparison for six treatment groups)	n.s.	<0.0001	<0.0001

SD = standard deviation; 5-ALA = 5-aminolevulinic acid; GC = combined chemotherapy of gemicitabine and cisplatin; *p* values are based on the comparison of the values at each treatment time point relative to the non-treated control group or GC alone group with using multiple comparison (the Kruskal-Wallis test) and the post hoc test (Dunn test).

**Table 4:** Comparison of post-treatment serum biomarkers among the treatment groups.

Treatment Group (*n* = 6 Per Group)	Serum Levels	Active Ghrelin (fmol/mL)	Deacyl Ghrelin (fmol/mL)	IL-6 (pg/mL)	IGF-1 (pg/mL)	Albumin (mg/mL)	Creatinine (mg/mL)
Non-treated control	Mean ± SD	4.3 ± 1.1	202 ± 122	76.8 ± 26.5	163 ± 36	0.99 ± 0.07	0.58 ± 0.07
Anamorelin	Mean ± SD	4.2 ± 1.8	234 ± 55	78.5 ± 17.5	144 ± 45	1.08 ± 0.15	0.59 ± 0.02
	*p* value (vs control)	n.s.	n.s.	n.s.	n.s.	n.s.	n.s.
5-ALA	Mean ± SD	3.9 ± 0.5	203 ± 92	59.2 ± 16.0	134 ± 18	0.92 ± 0.18	0.60 ± 0.18
	*p* value (vs control)	n.s.	n.s.	n.s.	n.s.	n.s.	n.s.
GC alone	Mean ± SD	2.0 ± 0.6	285 ± 93	54.8 ± 7.51	77 ± 25	0.98 ± 0.13	0.59 ± 0.02
	*p* value (vs control)	0.042	n.s.	n.s.	0.09	n.s.	n.s.
GC plus anamorelin	Mean ± SD	2.5 ± 0.4	263 ± 88	86.6 ± 36.0	195 ± 39	1.01 ± 0.3	0.54 ± 0.10
	*p* value (vs GC alone)	n.s.	n.s.	n.s.	0.034	n.s.	n.s.
GC plus 5-ALA	Mean ± SD	2.3 ± 0.8	248 ± 67	65.2 ± 21.4	187 ± 28	1.09 ± 0.08	0.57 ± 0.02
	*p* value (vs GC alone)	n.s.	n.s.	n.s.	n.s.	n.s.	n.s.
	*p* value (multiple comparison for six treatment groups)	0.039	n.s.	n.s.	0.025	n.s.	n.s.

SD = standard deviation; 5-ALA = 5-aminolevulinic acid; GC = combined chemotherapy of gemicitabine and cisplatin; IL-6 = interleukin-6; IGF-1 = Insulin-like growth factor-1; *p* values are based on the comparison of the values at each treatment time point relative to the non-treated control group or GC alone group with using multiple comparison (the Kruskal-Wallis test) and the post hoc test (Dunn test).

## Supplementary Materials

The following are available online at https://www.mdpi.com/2072-6694/12/7/1942/s1, Figure S1: Photograph of full blots in Western blot analysis for p-FOXO1 and actin, Table S1: Primary Antibodies, TaqMan gene expression primers, and ELISA kits used in this study.
