# Supplementary material for: Supplementary Oral Anamorelin Mitigates Anorexia and Skeletal Muscle Atrophy Induced by Gemcitabine Plus Cisplatin Systemic Chemotherapy in a Mouse Model
Source: Cancers (Basel). 2020 Jul 17;12(7):1942. doi: 10.3390/cancers12071942 (PMC7409153; doi:10.3390/cancers12071942)
Supplement: Supplementary file 1 [file cancers-12-01942-s001.pdf]

cancers

MDPI

*Article*

# Supplementary Oral Anamorelin Mitigates Anorexia and Skeletal Muscle Atrophy Induced by Gemcitabine Plus Cisplatin Systemic Chemotherapy in a Mouse Model

Makito Miyake, Shunta Hori, Yoshitaka Itami, Yuki Oda, Takuya Owari, Tomomi Fujii, Sayuri Ohnishi, Yosuke Morizawa, Daisuke Gotoh, Yasushi Nakai, Satoshi Anai, Kazumasa Torimoto, Nobumichi Tanaka and Kiyohide Fujimoto

## Supplementary Materials

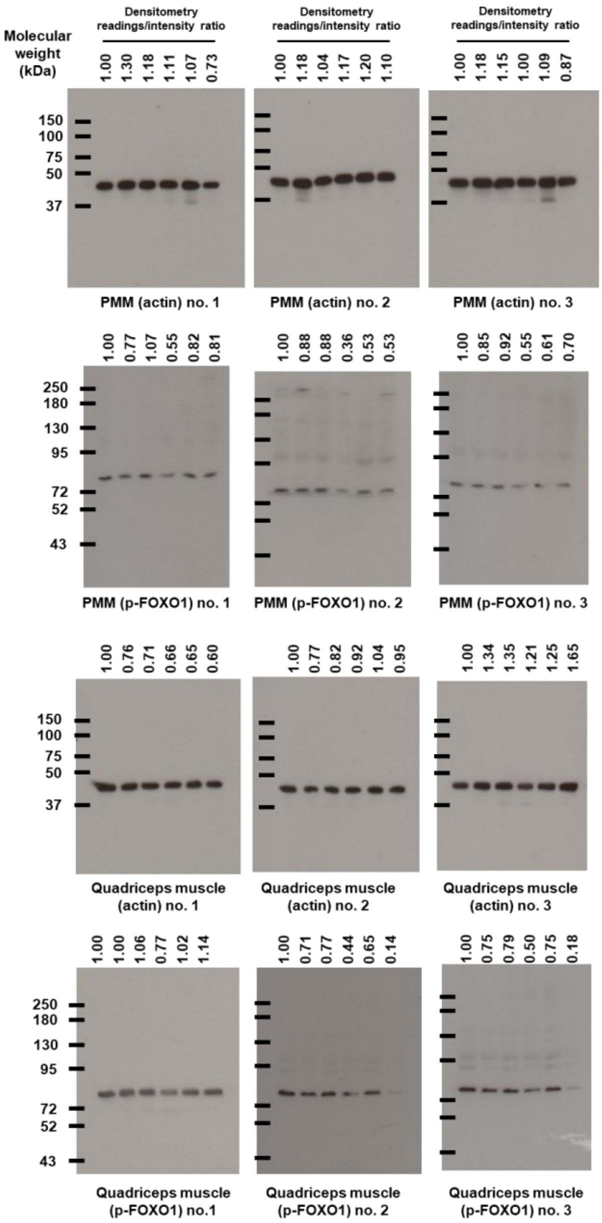


**Figure S1.** Photograph of full blots in Western blot analysis for p-FOXO1 and actin.

**Table S1.** Primary antibodies,TaqMan gene expression primers, and ELISA kits used in this study.

| Target Gene or Protein | Provider | ID or Catalogue Number | Application |
|---|---|---|---|
| phospho-FOXO1 | Novus Biologicals | NB100-81927 | Western blot (dilusion, 1:2000) |
| Actin | Sigma-Aldrich | clone AC-15 | Western, blot (dilusion 1:20,000) |
| MAFbx/Atrogin-1 | ThermoFisher scientific | Mm00499523_m1 | RT-PCR |
| MuRF1 | ThermoFisher scientific | Mm01185221_m1 | RT-PCR |
| Actin-beta | ThermoFisher scientific | Mm00607939_s1 | RT-PCR |
| Ghrelin | Abcam | ab129383 | Immunohistochemistry (dilusion, 1:5000) |
| Active ghrelin | SCETI, Tokyo, Japan | 97751 | ELISA |
| Deacyl ghrelin | SCETI, Tokyo, Japan | 97752 | ELISA |
| IL-6 | Proteintech Japan, Tokyo, Japan | KE10007 | ELISA |
| IGF-1 | Proteintech Japan, Tokyo, Japan | KE10032 | ELISA |
| Albumin | Cloud-Clone Corp, TX, USA | CEB028Mu | ELISA |
| Creatinine | Crystal Chem, IL, USA | 80350 | ELISA |

ELISA = enzyme-linked immunosorbent assay; FOXO1 = forkhead box protein O-1; RT-PCR = Reverse Transcription Polymerase Chain Reaction; MAFbx = muscle atrophy F-box; MuRF1 = muscle RING finger 1; IL-6 = interleukin-6; IGF-1 = Insulin-like growth factor-.
